# CD4 T Follicular Helper Cells and HIV Infection: Friends or Enemies?

**DOI:** 10.3389/fimmu.2017.00135

**Published:** 2017-02-20

**Authors:** Félicien Moukambi, Vasco Rodrigues, Yasmina Fortier, Henintsoa Rabezanahary, Chloé Borde, Bernard Krust, Guadalupe Andreani, Ricardo Silvestre, Constantinos Petrovas, Mireille Laforge, Jérôme Estaquier

**Affiliations:** ^1^Centre Hospitalier Universitaire (CHU) de Québec Research Center, Faculty of Medicine, Laval University, Québec, QC, Canada; ^2^CNRS FR3636, Faculty of Medecine des Saint-Pères, Paris Descartes University, Paris, France; ^3^School of Health Sciences, Life and Health Sciences Research Institute (ICVS), University of Minho, Braga, Portugal; ^4^ICVS/3B’s-PT Government Associate Laboratory, Braga/Guimarães, Portugal; ^5^Tissue Analysis Core, Vaccine Research Center, National Institute of Allergy and Infectious Diseases, National Institutes of Health, Bethesda, MD, USA

**Keywords:** AIDS, Tfh, CD4, B cell, vaccine, pathogen, SIV, reservoir

## Abstract

Follicular T helper (Tfh) cells, a subset of CD4 T lymphocytes, are essential for memory B cell activation, survival, and differentiation and assist B cells in the production of antigen-specific antibodies. Work performed in recent years pointed out the importance of Tfh cells in the context of HIV and SIV infections. The importance of tissue distribution of Tfh is also an important point since their frequency differs between peripheral blood and lymph nodes compared to the spleen, the primary organ for B cell activation, and differentiation. Our recent observations indicated an early and profound loss of splenic Tfh cells. The role of transcriptional activator and repressor factors that control Tfh differentiation is also discussed in the context of HIV/SIV infection. Because Tfh cells are important for B cell differentiation and antibody production, accelerating the Tfh responses early during HIV/SIV infection could be promising as novel immunotherapeutic approach or alternative vaccine strategies. However, because Tfh cells are infected during the HIV/SIV infection and represent a reservoir, this may interfere with HIV vaccine strategy. Thus, Tfh represent the good and bad guys during HIV infection.

Adaptive immunity against pathogens originates with the expansion of antigen-specific T lymphocytes in secondary lymphoid organs. T cells are a heterogeneous population (1). Based on an array of cell surface markers, distinct subsets have been discriminated including naive, central memory (T_CM_), effector memory (T_EM_), and terminally differentiated (T_DT_) T cells (2). The function of T_EM_ T cells is dependent not only on the production of cytokines, but also on the expression of a particular set of chemokine receptors that determine in a combinatorial fashion, the steps of extravasation and positioning in different tissue microenvironments (3–5). The discovery of follicular T helper (Tfh) cells dates back to the early 1990s, during a key period coincident with the acknowledgment of the crucial importance of chemokines in immunology. CXCL13 or B cell-attracting chemokine 1 (BCA-1) (6, 7) is the selective chemokine ligand for CXC chemokine receptor 5 (CXCR5, originally named MDR15/BLR1); the phenotypic marker used to characterize Tfh cells in early studies (8, 9).

Circulating memory CD4 T cells bearing the phenotype of Tfh cells have been termed “circulating Tfh” or “peripheral Tfh.” While some assume that peripheral Tfh cells are the *bona fide* circulating counterparts of lymphoid tissue Tfh cells (10, 11), such notion remains controversial (12) as revealed by RNA sequencing (13) and levels of programmed death molecule-1 (PD-1) (14, 15) in circulating Tfh cells compared to those in lymphoid tissues (16). Tfh cells are relatively scarce in peripheral blood of healthy individuals. Therefore, it is of crucial importance to analyze Tfh cells in deep tissues.

Because of their ability to support the generation of strong antibody responses, memory Tfh cells are the subject of intense investigation aimed at harnessing this property for novel vaccination approaches as well as immune therapies for infectious diseases and cancer. Growing researches have been dedicated to the characterization of Tfh dynamics during microbe infections, particularly during HIV. This review summarizes recent advances in this growing field.

## Dynamics of Tfh Cells During AIDS

Lymphopenia is a hallmark of the progression to AIDS. As infection progresses, CD4 T cell count progressively declines. The excessive induction of apoptosis and immune activation has been proposed as major mechanisms responsible for the CD4 T cell depletion (17, 18). Studies performed in pathogenic and non-pathogenic lentiviral infections in non-human primate models have further suggested a correlation between the pathology and the levels of CD4 T cells apoptosis and immune activation (19–21). The extent of T cells apoptosis in lymph nodes (LNs) during primary infection predicts disease progression (22, 23) and increased apoptosis is also observed in the intestinal lamina propria (24, 25). In particular, memory CD4^+^ T cells are rapidly depleted in lymphoid tissues (26, 27) and are more prone to undergo apoptosis (23, 28).

As a subset of memory CD4 T cells, Tfh cells were expected to undergo progressive depletion during AIDS. However, Tfh frequencies are increased in the blood (29), and LNs of chronically infected individuals (30). This frequency increases among the pool of memory CD4 T cells in SIV-infected monkeys (31–33). On the contrary, Boswell *et al*. (13) showed a loss of Tfh cells during HIV infection. Petrovas *et al*. (34) have initially reported that half of the chronically SIV-infected rhesus macaques (RM) had increased numbers of LN Tfh cells, which are associated with preserved lymphoid architecture and lower accumulation of naive CD4 T cells, a hallmark of non-progression to AIDS. Two recent reports also indicated that the numbers of Tfh are higher in LNs of non-progressor compared to progressor SIV-infected RMs (35, 36). While the spleen contains the majority of Tfh cells, their dynamics in this compartment was still missing. We recently demonstrated an early depletion of splenic Tfh cells after SIV infection of RMs (16). This depletion persists in monkeys progressing faster to AIDS. These results underline the critical impact of tissue compartmentalization on Tfh cell dynamics during AIDS. Therefore, assuming that the dynamics of circulating Tfh reflects the dynamics of their lymphoid tissue counterparts should be taken with caution and merits to be reevaluated.

## Transcriptional Factors and Abnormal Differentiation of Tfh Cells During AIDS

**Figure 1:**
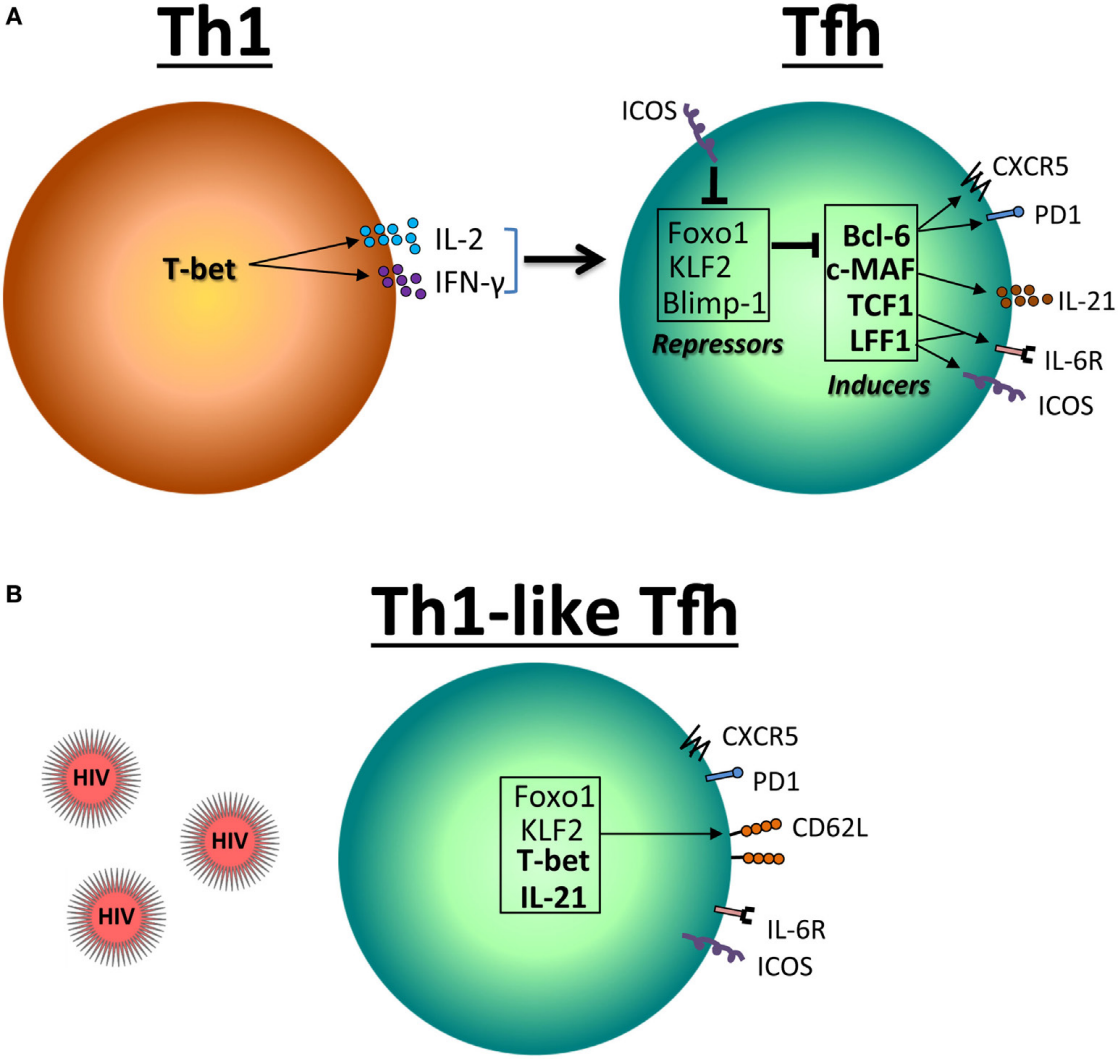
**Reciprocal expression of transcriptional factors in Th1 and follicular T helper (Tfh) cells**. **(A)** T-bet is the principal transcription factor for the differentiation and function of Th1 CD4 T cell. T-bet inhibits the expression of programmed death molecule-1 (PD-1) but induces IL-2 and IFN-γ, which in turn leads to the expression of Foxo1 and Krüppel-like factor 2 (KLF2). These factors including Blimp-1 inhibit Bcl-6, c-MAF, TCF1, and LEF1 necessary for the differentiation and function of Tfh cells. **(B)** In the context of HIV/SIV infection, a Th1-like Tfh profile is associated with the expression of T-bet.

**Figure 2:**
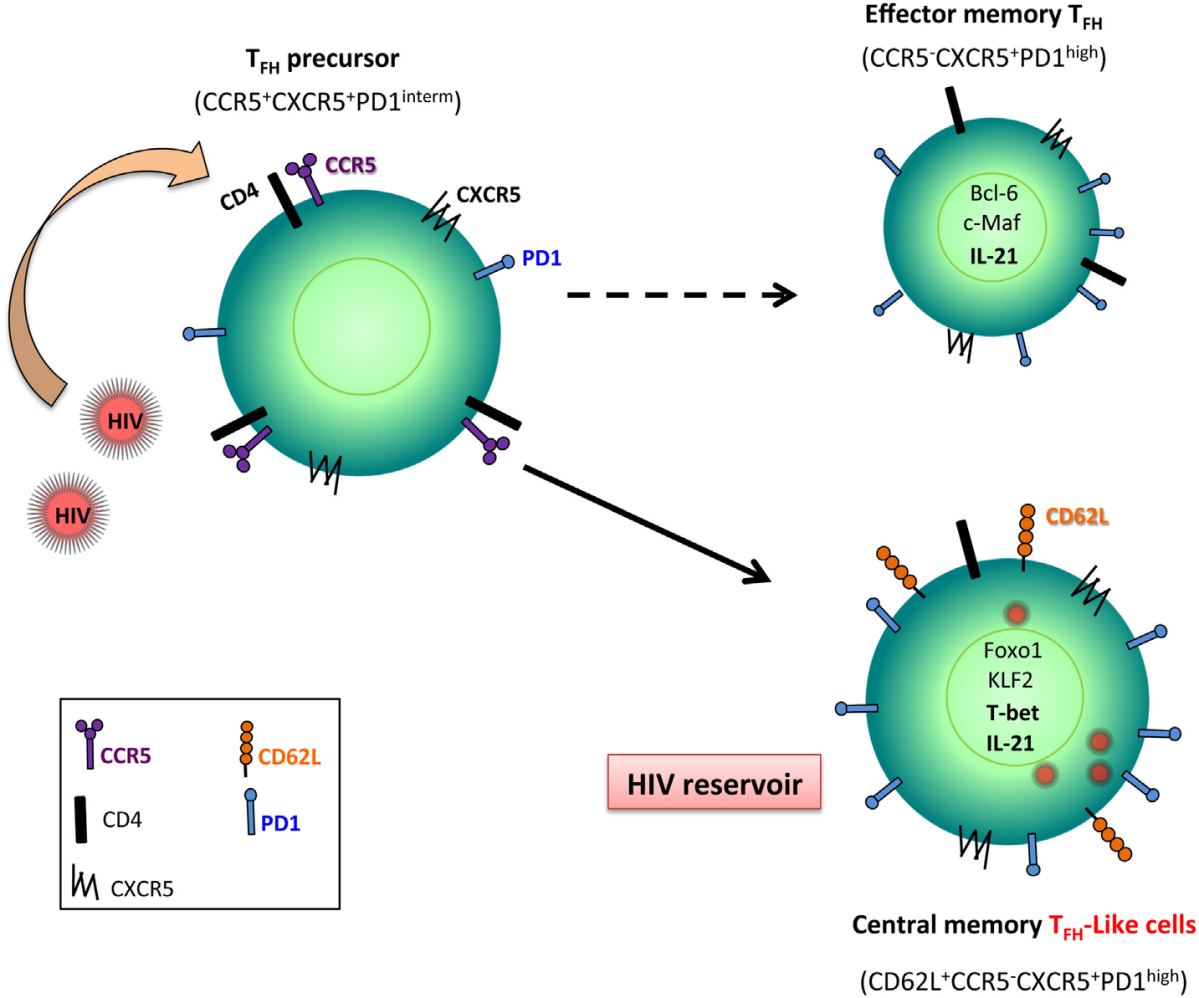
**Follicular T helper (Tfh) cell, a reservoir for HIV**. Tfh precursor cells that express CCR5, the main co-receptor for HIV/SIV entry, are early infected. Because Foxo1 and Krüppel-like factor 2 (KLF2) are upregulated in Tfh cells during HIV/SIV infection, these transcriptional factors control the full maturation of Tfh leading to central memory cells associated with the expression of CD62L. Because these cells are less sensitive to undergo death than effector memory T cells, infected Tfh cells represent potent reservoirs for viral replication.

Bcl-6 promotes the Tfh transcriptional program, at least in part by suppressing the expression of the transcriptional regulators such as T-bet (Th1) (37), RORγt (Th17) (38), GATA3 (Th2) (39), and Blimp-1 (40–42). Bcl-6 and Blimp-1 are mutually antagonistic, and the balance between the expression of these two factors is a critical element in determining the fate of Tfh cells. Nevertheless, others have proposed an alternative, STAT3-independent pathway, for Tfh cell development (43). In addition to Bcl-6, it has been shown that Maf plays an important role in the differentiation and/or function of Tfh cells (44, 45). Among the transcriptional repressors, Krüppel-like factor 2 (KLF2) restrains Tfh cell differentiation by inhibiting CXCR5 and Bcl-6 expression (46, 47) (Figure 1A). KLF2 is one of the genes targeted by Foxo1, which has been also shown to negatively regulate the differentiation of Tfh cells through at least the involvement of the E3 ubiquitin ligase Itch (48, 49). KLF2 as well as Foxo1 regulate the expression of CD62L (50, 51), which may have an impact on T cell redistribution. Whereas in uninfected mice, most of Tfh cells are T_EM_ cells (CD45RA^−^CD62L^−^), they exhibit a central T_EM_ phenotype (CD45RA^−^CD62L^+^) after lymphocytic choriomeningitis virus infection (12). Our results demonstrated similar commutation of Tfh splenocytes during SIV infection (16). Because T_CM_ cells are less prone to die than T_EM_ CD4 T cells of HIV- and SIV-infected individuals (23, 28, 52–54), the observation that splenic Tfh cells of SIV-infected RMs present a switch toward T_CM_ phenotype may reconcile the apparently contradictory observations that the frequency of Tfh cells increases among the pool of memory CD4 T cells, whereas total Tfh cell numbers decreased. Our results have also indicated that Tfh transiently expressed higher levels of Bcl-6 and Maf, whereas Foxo1 and KLF2 are increased in Tfh cells of SIV-infected RMs concomitantly with higher levels of CD62L (16) (Figures 1B and 2). However, the list of transcriptional factors regulating Tfh cell differentiation is growing, which includes the basic leucine zipper transcriptional ATF-like (BATF), interferon regulatory factor 4, achaete–scute complex homolog 2 (55), NFATC1 (56), STAT1 (57), TCF1 (58–61), and Bob1 (62), and merit to be further analyzed in the context of AIDS.

Our analyses have also indicated higher T-bet expression in splenic Tfh cells at the chronic phase indicating the accumulation of Th1-like Tfh cells (16) (Figures 1B and 2). Interestingly, from these initial observations the list of pathogens impacting on Tfh function and differentiation leading to abortive differentiation is growing (63–66). Although T-bet has been reported to antagonize the expression of IL-21 (67, 68), IL-21 mRNA expression in sorted splenic Tfh cells of chronically SIV-infected RMs is not lower as compared to splenic Tfh cells isolated from healthy monkeys. The depletion Tfh cells may participate in the decrease of IL-21 that has been reported in HIV-infected individuals (69, 70). Such observation is of crucial importance, given the known role of IL-21 in controlling chronic viral infections by supporting CD8 T cell function (71–73). Schultz *et al*. (74) proposed that expression of IL-21 can be a surrogate marker for Tfh cells that can be used in various clinical settings as a useful monitoring tool for immune-based interventions aimed at selectively boosting Tfh cell function in humans (74). However, this should be extremely limitative in the sense that IL-21 would be therefore enough to mimic Tfh cell function, not integrating the role of cell–cell contact interaction and the architecture of lymphoid organs.

## Tfh Cells and B Cell Immunity During AIDS

Besides CXCR5 and high levels of PD-1, Tfh cells express the inducible T-cell costimulator (ICOS) and CD40L (57, 75). Thus, Tfh cells provide survival and proliferation signals to B cells *via* CD40L, ICOS, IL-21, and BATF, which compete with death-inducing Fas–FasL interactions (76–78). IL-21 production by Tfh cells is an important mediator in most processes occurring inside germinal centers (GCs), namely, affinity maturation, class-switching, and differentiation of long-lived plasmacytoid cells. The depletion of Tfh cells in the spleen very early after infection may participate in the absence of maturation and loss of memory B cells (79–81). We found a positive correlation between B cell differentiation and Tfh cell number in the spleen of SIV-infected RMs (16), but no correlation between the extent of infection of Tfh cells and the percentages of memory B cell subsets, suggesting that infection of Tfh cells is not directly associated with abnormal B cell differentiation (16). Cubas and colleagues have proposed that excessive and persistent triggering of PD-1 on LN Tfh cells may affect their ability to provide adequate B cell help (31). It is noteworthy that patients who are responders to a Flu vaccine display an expansion of circulating Tfh-like cells compared to non-responders (82), supporting a role of Tfh cells in maintaining the pool of long live memory B cells (36). It has been proposed in HIV-infected individuals that a subpopulation of peripheral blood memory PD-1^+^CXCR5^+^CD4^+^ T cells is associated with the development of broadly neutralizing antibodies (bnAbs) (83). In the sera, higher level of CXCL13, the CXCR5 ligand, is associated with the detection of bnAbs-positive in HIV-infected individuals (84). They propose that individuals able to generate HIV bnAbs may have superior GC responses (84). On the contrary, defect in Tfh cells can be associated with hypergammaglobulinemia and the absence of bnAbs. Therefore, the early depletion of Tfh cells in the spleen of SIV-infected monkeys may contribute to the absence of efficient B cell immune response in controlling HIV and SIV infections. The significant association between frequency and quality (IFN-γ^low^IL-21^high^) of Env-specific Tfh cells and development of broad neutralization activity was recently described in NHP infected with SHIV virus (36). The co-evolution of virus (a process likely affected by the immunological pressure of the humoral responses too) and Tfh responses could represent major biological factors underlying the development of bnAbs. Investigation of the follicular immune reactions in lymph nodes from patients mounting bnAbs combined with studies utilizing the NHP model could provide critical information regarding the relative impact of these factors on this process.

Furthermore, several studies indicate that full expression of the Tfh differentiation program depends on cognate interactions between primed CD4 T cells and antigen-activated B cells (40, 85). Thus, a reciprocal regulation exists between Tfh and GC B cells, mediated by ICOS–ICOSL and CD40–CD40L interactions (86). In mice, the absence of PD-1 impairs Tfh function, resulting in suboptimal synthesis of important cytokines for the differentiation of long-lived plasma B cells (87). In SIV-infected RMs, B cell follicles and GCs become barely distinguishable in progressor animals, but are preserved in non-progressors, highlighting the profound remodeling of the normal splenic architecture that occurs during progression to AIDS (16). Tfh cells are hardly detectable on the B cell follicles of the spleen and LNs (16, 88, 89).

Altogether, these observations showing abortive differentiation (quality) associated with the loss of Tfh cells (quantity) provides rationale for interventions aimed at boosting Tfh cell responses in the setting of HIV prevention or therapy, in particular for inducing the generation of more efficient antibodies and bnAbs.

## Infection of Tfh Cells

Virus production in human immunodeficiency virus 1-infected individuals is largely the result of a dynamic process involving continuous rounds of *de novo* infection and replication in CD4 T cells with rapid turnover of both free virus and virus-producing cells. Thus, the level of viral load in the peripheral blood is a strong predictor of disease progression in pathogenic lentivirus infection (90–92). Earlier it has been clearly shown that even during clinical latency, HIV infection is never completely silent (93). Productively infected cells are detected at a higher frequency, emphasizing the progressive nature of HIV infection in lymphoid organs. Peripheral lymphoid tissues such as axillary and inguinal (LNs) and the spleen are major sites for HIV/SIV replication. An increasing body of evidence suggests that reservoirs, cell types or anatomical sites (“sanctuaries”), represent a major barrier to virus eradication (94). This has been recently demonstrated by the observation that despite intense ART therapy introduced early after infection, drug regimen has been unable to clear reservoirs (95). In this context, intestine tissues and their draining LNs also represent likely sanctuaries for persistent viral replication due to the particularity of the immune response in these sites, which are exposed to myriad of antigens to surveil the intestinal microbiome (96).

In the context of natural infection, it was clearly established that productively infected cells and virus trapped at the follicular dendritic cell (FDC) surface, showing a diffuse labeling over the FDC network in GC, are detectable in lymphoid tissues. The amount of viral particles trapped in the region of GCs increases with the pathogenicity (97, 98). Trapping of SIV in GCs is also observed in non-pathogenic SIV-infected African green monkeys (AGMs) (21) or in sooty mangabeys at the border of the GCs where Tfh cells are localized (99). During the early acute phase of infection, the viral dynamics in peripheral blood is quite similar between pathogenic and non-pathogenic lentiviral infections. However, a major distinction is evident by the end of the acute phase with higher numbers of SIV RNA^+^ cells in RMs compared to AGMs, in which productively infected cells are barely detectable (21, 100). In HIV long-term non-progressors, it has been recently reported that B cell follicles represent an active site for viral replication (33), suggesting distinct viral dynamics. Furthermore, a clear difference in the dynamics of GC and B cells is observed between non-pathogenic (AGM) and pathogenic (RM) lentiviral infections. SIV-infected AGMs showed a more prominent B-cell activation than SIV-infected RMs, as manifested by the level of Ki67+ cells in the LN GCs at the set point compared to that in RMs (21, 101, 102). Altogether these observations indicated that the dynamic of GC and innate immunity is inversely correlated with viral replication and pathogenicity in peripheral LNs (100).

Growing evidences suggest that Tfh cells are infected by HIV/SIV early after infection (33, 89, 103–105). Splenic Tfh cells are infected early after SIV inoculation in RMs. Importantly, the frequencies and total numbers of SIV DNA^+^ Tfh cells were higher at the chronic phase in non-progressor than in progressor RMs (16) suggesting that this population may be a latent pool associated with a “silent” Tfh phenotype in non-progressors. Consistent with *in situ* hybridization, few SIV p28 positive cells are observed in follicles of LN GCs of non-progressors (106).

Because Tfh cells do not express CCR5, the main co-receptor for HIV and SIV, how to explain that this memory subset is infected? Circulating Tfh cells are more permissive *in vitro* to HIV infection than non-Tfh cells (107). It has been reported that Tfh precursor cells (LN CXCR5^+^PD-1^int^CD4^+^) express CCR5 (106). This observation suggests that this subset (PD-1^int^CD4^+^) can be the target of infection and not Tfh themselves (Figure 2). Furthermore, the observation that Tfh cells display a T_CM_ phenotype (16) may favor viral persistence because this CD4 T cell subset is less potent to die than T_EM_ CD4 T cell subset. Our results have also demonstrated that despite their high frequency in SIV DNA, Tfh cells of non-progressors showed a similar or lower level of cellular SIV RNA compared to progressors (16), pointing to the fact that non-progressor Tfh cells might be less active to replicate SIV than Tfh cells of progressors, which might be related to the differentiation stage of Tfh cells (central *versus* T_EM_ subset). Interestingly, HIV long terminal repeat contains binding sites for Bcl-6 that has previously been reported to repress HIV transcription (108), which may control HIV replication in Tfh cells.

Altogether these results indicate that Tfh cells may represent a potent viral reservoir in lymphoid tissues, in particular in non-progressors.

## Conclusion

Although this review synthesizes recent advances on the role of Tfh cells in the context of HIV/SIV infections, several key questions remain to be addressed. By which mechanisms Tfh are early lost? Which are the processes leading to abortive Tfh cell differentiation by inducing a Th1-like profile? Does preventing CD4 T cell depletion boost the generation of high affinity and HIV-neutralizing Abs? Does ART therapy improve the quality and the quantity of splenic Tfh cells? Therefore, understanding the biology and dynamics of Tfh cells in deep tissues is of crucial interest for the development of novel vaccine strategies and the delineation of the cellular and molecular mechanisms leading to the formation of persistent reservoirs for HIV.

## Author Contributions

FM, VR, YF, HR, CB, BK, GA, RS, CP, ML, and JE contributed to writing of this review.

## Conflict of Interest Statement

The authors declare that the research was conducted in the absence of any commercial or financial relationships that could be construed as a potential conflict of interest.
